# Protein kinase A enhances lipopolysaccharide-induced IL-6, IL-8, and PGE_2 _production by human gingival fibroblasts

**DOI:** 10.1186/1477-5751-11-10

**Published:** 2012-03-27

**Authors:** Toshiaki Ara, Yoshiaki Fujinami, Hiroko Urano, Kaname Hirai, Toshimi Hatori, Hiroo Miyazawa

**Affiliations:** 1 Department of Pharmacology, Matsumoto Dental University, 1780 Gobara Hirooka, Shiojiri, Nagano 399-0781, Japan; 2 Institute for Oral Science, Matsumoto Dental University, 1780 Gobara Hirooka, Shiojiri, Nagano 399-0781, Japan; 3 Department of Oral Microbiology, Matsumoto Dental University, 1780 Gobara Hirooka, Shiojiri, Nagano 399-0781, Japan; 4 Department of Oral Health Promotion, Graduate School of Oral Medicine, Matsumoto Dental University, 1780 Gobara Hirooka, Shiojiri, Nagano 399-0781, Japan

**Keywords:** Protein kinase A, interleukin, prostaglandin E_2_, human gingival fibroblast

## Abstract

**Objective:**

Periodontal disease is accompanied by inflammation of the gingiva and destruction of periodontal tissues, leading to alveolar bone loss in severe clinical cases. Interleukin (IL)-6, IL-8, and the chemical mediator prostaglandin E_2 _(PGE_2_) are known to play important roles in inflammatory responses and tissue degradation.

Recently, we reported that the protein kinase A (PKA) inhibitor H-89 suppresses lipopolysaccharide (LPS)-induced IL-8 production by human gingival fibroblasts (HGFs). In the present study, the relevance of the PKA activity and two PKA-activating drugs, aminophylline and adrenaline, to LPS-induced inflammatory cytokines (IL-6 and IL-8) and PGE_2 _by HGFs were examined.

**Methods:**

HGFs were treated with LPS from *Porphyromonas gingivalis *and H-89, the cAMP analog dibutyryl cyclic AMP (dbcAMP), aminophylline, or adrenaline. After 24 h, IL-6, IL-8, and PGE_2 _levels were evaluated by ELISA.

**Results:**

H-89 did not affect LPS-induced IL-6 production, but suppressed IL-8 and PGE_2 _production. In contrast, dbcAMP significantly increased LPS-induced IL-6, IL-8, and PGE_2 _production. Up to 10 μg/ml of aminophylline did not affect LPS-induced IL-6, IL-8, or PGE_2 _production, but they were significantly increased at 100 μg/ml. Similarly, 0.01 μg/ml of adrenaline did not affect LPS-induced IL-6, IL-8, or PGE_2 _production, but they were significantly increased at concentrations of 0.1 and 1 μg/ml. In the absence of LPS, H-89, dbcAMP, aminophylline, and adrenaline had no relevance to IL-6, IL-8, or PGE_2 _production.

**Conclusion:**

These results suggest that the PKA pathway, and also PKA-activating drugs, enhance LPS-induced IL-6, IL-8, and PGE_2 _production by HGFs. However, aminophylline may not have an effect on the production of these molecules at concentrations used in clinical settings (8 to 20 μg/ml in serum). These results suggest that aminophylline does not affect inflammatory responses in periodontal disease.

## Background

Periodontal disease is accompanied by inflammation of the gingiva and destruction of periodontal tissues, leading to alveolar bone loss in severe clinical cases. Interleukin (IL)-6, IL-8, and prostaglandin E_2 _(PGE_2_) are known to play important roles in inflammatory responses and tissue degradation. IL-6 has the ability to induce osteoclastogenesis [1,2]. IL-8 acts as a chemoattractant for neutrophils [3] that produce proteases such as cathepsin, elastase, or matrix metalloproteinase (MMP)-8, leading to tissue degradation. PGE_2 _has several functions in vasodilation, the enhancement of vascular permeability and pain, and the induction of osteoclastogenesis, and is believed to play important roles in inflammatory responses and alveolar bone resorption in periodontal disease [4].

Recently, we reported that the protein kinase A (PKA) inhibitor H-89 suppresses LPS-induced IL-8 production by human gingival fibroblasts (HGFs) [5]. This finding suggests that the PKA pathway enhances LPS-induced IL-8 production, and that taking PKA-activating drugs results in an increase of inflammatory cytokines production, and further, the exacerbation of periodontal disease. PKA-activating drugs are xanthine derivatives and β-adrenergic agonists. Xanthine derivatives such as theophylline and aminophylline inhibit the cAMP-degrading enzyme phosphodiesterase (PDE), increase intracellular cAMP, and then activate the PKA pathway [6]. These drugs are clinically used as cardiotonic agents for cardiac failure or as bronchodilator agents for chronic obstructive pulmonary disease. In contrast, β-adrenergic agonists activate adenylate cyclase, increase intracellular cAMP, and then activate the PKA pathway. Because withdrawal of these drugs, in particular xanthine derivatives, leads to exacerbation of cardiac failure and chronic obstructive pulmonary disease, withdrawal is difficult in patients having these diseases. For these reasons, there is a need to examine whether these drugs affect the inflammatory responses in periodontal disease.

HGFs are the most prominent cells in periodontal tissue. And LPS-treated HGFs produce inflammatory cytokines such as IL-6 and IL-8, and inflammatory chemical mediators such as PGE_2 _[1,7,8].

Moreover, because HGFs have sustained production of IL-6 and IL-8 [9] and PGE_2 _[10] in the presence of LPS, these mediators and cytokines in periodontal tissues are thought to be derived from HGFs.

Therefore, we believe that examining the effects of these drugs on HGFs, as well as on monocytes and macrophages, is important in the study of periodontal disease. In the present study, we examined the relevance of the PKA activity, and of drugs activating the PKA pathway (aminophylline and adrenaline), to LPS-induced IL-6, IL-8, and PGE_2 _production by HGFs.

## Results

### Relevance of the PKA activity to LPS-induced IL-6, IL-8, and PGE_2 _production

**Figure 1:**
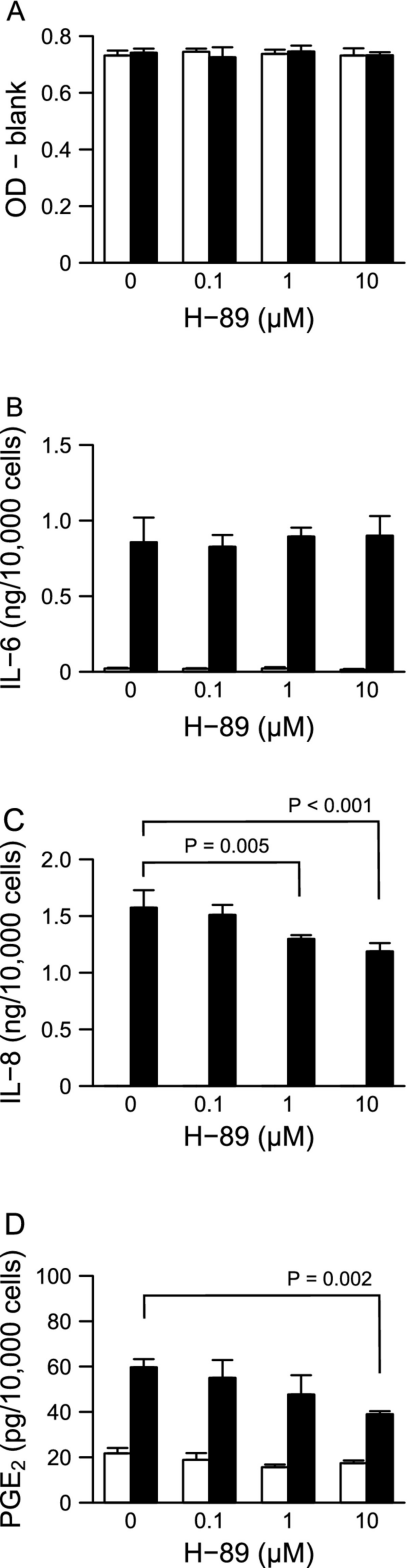
**Relevance of PKA inhibition to HGFs viability and the production of IL-6, IL-8, and PGE_2_**. HGFs were treated with combinations of LPS (0 and 10 ng/ml) and H-89 (0, 0.1, 1, and 10 μM) for 24 h. (**A**) Viable cell numbers were measured using WST-8 and are expressed as OD-blank (mean ± S.D., n = 3). (**B-D**) The concentrations of IL-6, IL-8, and PGE_2 _were measured by ELISA. The concentrations were adjusted by the viable cell numbers (**A**) and are expressed as ng or pg per 10,000 cells (mean ± S.D., n = 3). (**B**) IL-6, (**C**) IL-8, and (**D**) PGE_2_. Open bars, treatment without LPS; closed bars, treatment with 10 ng/ml of LPS. P values were calculated by the pairwise comparison test corrected with the Holm method (6 null hypotheses).

First, we examined whether the PKA inhibitor H-89 affects the production of inflammatory cytokines (IL-6 and IL-8) and PGE_2 _by HGFs. Because our previous report has shown that H-89 suppresses LPS-induced IL-8 production [5], we used this result as control in this study. H-89 showed little or no effect on cell viability in the absence or presence of LPS at 24 h after stimulation (Figure 1A). In the absence of LPS, H-89 did not affect IL-6, IL-8, or PGE_2 _production (Figure 1B-D). When HGFs were treated with 10 ng/ml of LPS, HGFs produced large amounts of IL-6, IL-8, and PGE_2_. Up to 10 μM of H-89 did not affect LPS-induced IL-6 production, but decreased IL-8 and PGE_2 _production (Figure 1B-D).

**Figure 2:**
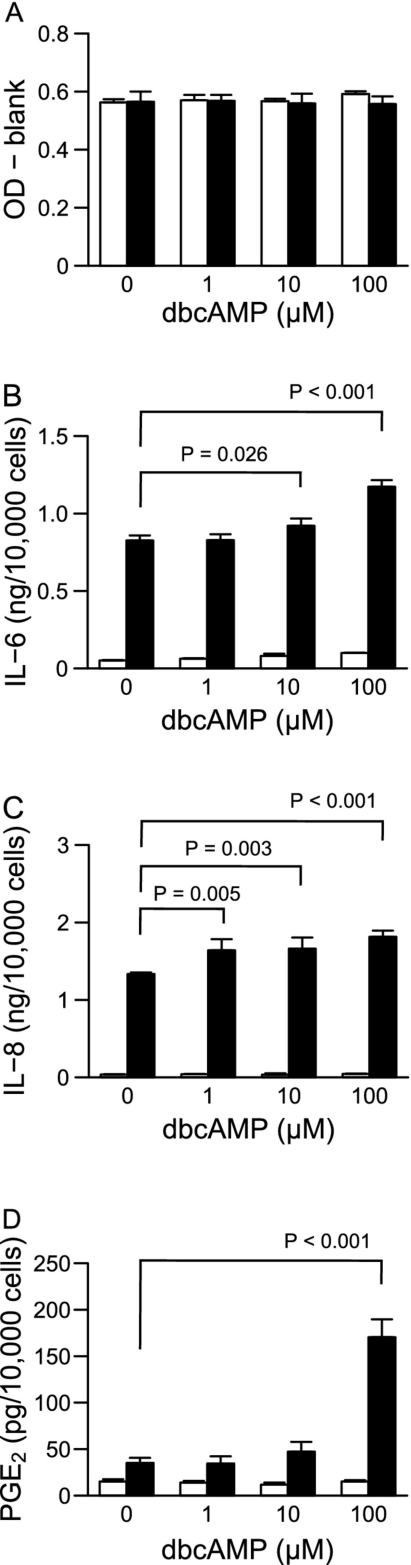
**Relevance of PKA activation to HGFs viability and the production of IL-6, IL-8, and PGE_2_**. HGFs were treated with combinations of LPS (0 and 10 ng/ml) and dbcAMP (0, 1, 10, and 100 μM) for 24 h. (**A**) Viable cell numbers were measured using WST-8 and are expressed as OD-blank (mean ± S.D., n = 3). (**B-D**) The concentrations of IL-6, IL-8, and PGE_2 _were measured by ELISA. The concentrations were adjusted by the viable cell numbers (**A**) and are expressed as ng or pg per 10,000 cells (mean ± S.D., n = 3). (**B**) IL-6, (**C**) IL-8, and (**D**) PGE_2_. Open bars, treatment without LPS; closed bars, treatment with 10 ng/ml of LPS. P values were calculated by the pairwise comparison test corrected with the Holm method (6 null hypotheses).

Next, we examined whether the cAMP analog dbcAMP affects the production of IL-6, IL-8, and PGE_2 _by HGFs. dbcAMP also showed little or no effect on cell viability in the absence or presence of LPS at 24 h after stimulation (Figure 2A). In the absence of LPS, dbcAMP did not affect IL-6, IL-8, or PGE_2 _production (Figure 2B-D). In contrast, dbcAMP significantly increased LPS-induced IL-6, IL-8, and PGE_2 _production in a dose-dependent-manner (Figure 2B-D).

### Relevance of drugs that activate the PKA pathway to LPS-induced IL-6, IL-8, and PGE_2 _production

**Figure 3:**
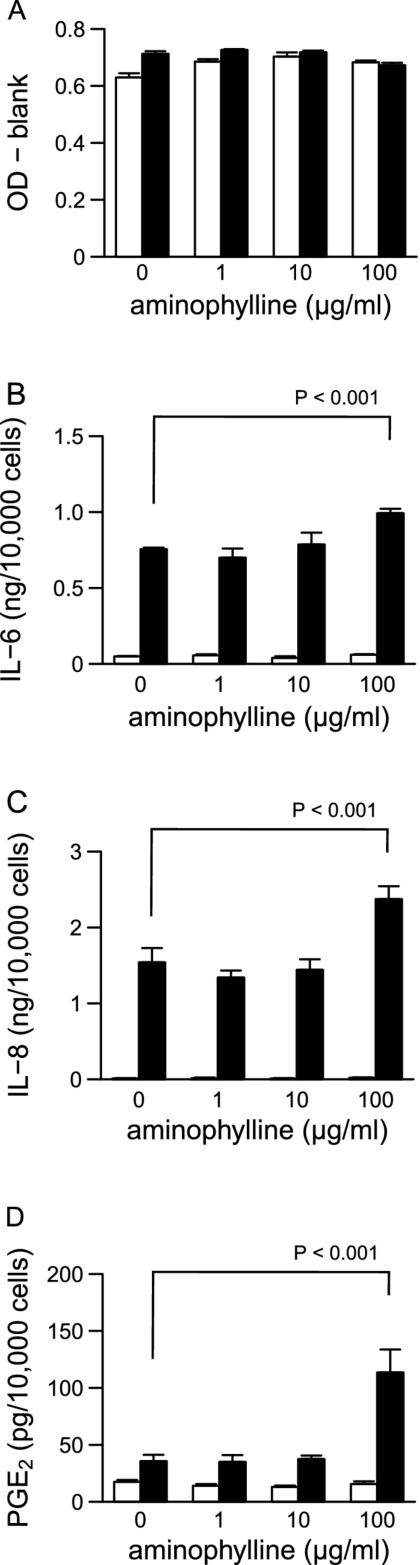
**Relevance of aminophylline to HGFs viability and the production of IL-6, IL-8, and PGE_2_**. HGFs were treated with combinations of LPS (0 and 10 ng/ml) and aminophylline (0, 1, 10, and 100 μg/ml) for 24 h. (**A**) Viable cell numbers were measured using WST-8 and are expressed as OD-blank (mean ± S.D., n = 3). (**B-D**) The concentrations of IL-6, IL-8, and PGE_2 _were measured by ELISA. The concentrations were adjusted by the viable cell numbers (**A**) and are expressed as ng or per 10,000 cells (mean ± S.D., n = 3). (**B**) IL-6, (**C**) IL-8, and (D) PGE_2_. Open bars, treatment without LPS; closed bars, treatment with 10 ng/ml of LPS. P values were calculated by the pairwise comparison test corrected with the Holm method (6 null hypotheses).

**Figure 4:**
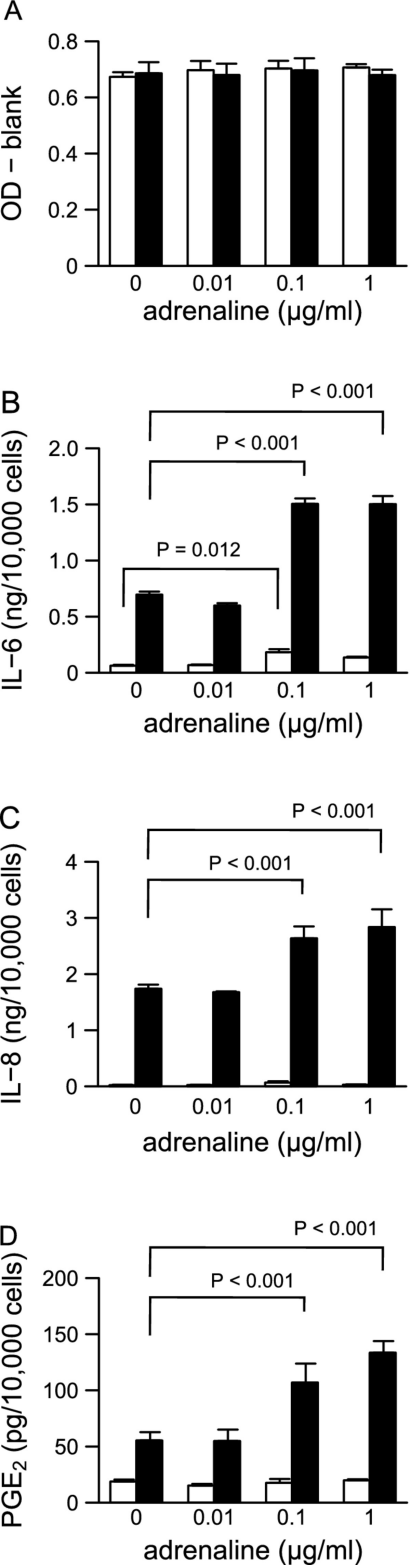
**Relevance of adrenaline to HGFs viability and the production of IL-6, IL-8, and PGE_2_**. HGFs were treated with combinations of LPS (0 and 10 ng/ml) and adrenaline (0, 0.01, 0.1, and 1 μg/ml) for 24 h. (**A**) Viable cell numbers were measured using WST-8 and are expressed as OD-blank (mean ± S.D., n = 3). (**B-D**) The concentrations of IL-6, IL-8, and PGE_2 _were measured by ELISA. The concentrations were adjusted by the viable cell numbers (**A**) and are expressed as ng or pg per 10,000 cells (mean ± S.D., n = 3). (**B**) IL-6, (**C**) IL-8, and (**D**) PGE_2_. Open bars, treatment without LPS; closed bars, treatment with 10 ng/ml of LPS. *P *values were calculated by the pairwise comparison test corrected with the Holm method (6 null hypotheses).

Because dbcAMP enhanced LPS-induced IL-6, IL-8, and PGE_2 _production, we examined the effects of aminophylline and adrenaline, which both increase intracellular cAMP concentrations and activate the PKA pathway, on IL-6, IL-8, and PGE_2 _production. Both aminophylline and adrenaline showed little or no effect on cell viability in the absence or presence of LPS (Figure 3A and 4A). In the absence of LPS, aminophylline and adrenaline did not affect IL-6, IL-8, or PGE_2 _production (Figure 3B-D and 4B-D). Up to 10 μg/ml of aminophylline did not affect LPS-induced IL-6, IL-8, or PGE_2 _production, but they were significantly increased at 100 μg/ml (Figure 3B-D). Similarly, 0.01 μg/ml of adrenaline did not affect LPS-induced IL-6, IL-8, or PGE_2 _production, but they were significantly increased at concentrations of 0.1 and 1 μg/ml (Figure 4B-D).

## Discussion

In the present study, we examined the relevance of the PKA activity to LPS-induced IL-6, IL-8, and PGE_2 _production. We showed that the PKA inhibitor H-89 suppressed LPS-induced IL-8 and PGE_2 _production, and that the PKA activator dbcAMP enhanced LPS-induced IL-6, IL-8, and PGE_2 _production. Moreover, we showed that aminophylline and adrenaline enhanced this production. However, H-89, dbcAMP, aminophylline, and adrenaline had no relevance to the production of these molecules in the absence of LPS. These results suggest that the PKA pathway enhances the LPS signaling pathway in HGFs, but that the PKA pathway alone does not enhance LPS-induced IL-6, IL-8, and PGE_2 _production.

Several reports have demonstrated the relevance of LPS to the PKA activity. LPS enhances cAMP accumulation in a mouse pituitary cell line [11] and in human bladder epithelial cells [12]. Moreover, LPS enhances cAMP response element binding protein (CREB) phosphorylation and its DNA-binding ability, and these activities are inhibited by H-89 [13]. Although there is no comparable report for HGFs, a similar mechanism may be present.

Additionally, various reports have shown the relevance of the PKA activity to IL-6, IL-8, and PGE_2 _production. However, whether the PKA pathway enhances or suppresses their production is different in depending on the cell type and genes in question. (1) The PKA pathway enhances LPS-induced IL-6 production. For instance, calcitonin gene-related peptide, which increases cAMP accumulation, enhances LPS-induced IL-6 production in mouse peritoneal macrophages [14], and H-89 suppresses IL-6 expression in human blood monocytes [15]. In the present study, dbcAMP, aminophylline, and adrenaline enhanced LPS-induced IL-6 production by HGFs to the same extent as by monocytes and macrophages in previous reports [14,15]. In contrast, H-89 had no relevance to LPS-induced IL-6 production. The reasons for this discrepancy remain to be elucidated. However, we assume that the enhancement of PKA activity by LPS treatment is weak in HGFs. (2) The relevance of the PKA activity to IL-8 production are different among several previous reports. The PKA pathway suppresses LPS-induced IL-8 production, *i.e*. β-adrenergic agonists and dbcAMP suppress and H-89 enhances LPS-induced IL-8 production by human monocytic THP-1 cells [16]. In contrast, cAMP analogs have been shown to have no relevance to LPS-induced IL-8 production by human macrophages [17] and by human peripheral blood mononuclear cells [18]. Our results showing that the PKA pathway enhanced LPS-induced IL-8 production by HGFs is different from these previous reports, and therefore may be an important characteristic of HGFs. (3) The PKA pathway enhances LPS-induced PGE_2 _production. dbcAMP enhanced LPS-induced cyclooxygenase-2 (COX-2) expression and PGE_2 _production by human blood moncytes [19] and mouse RAW264.7 macrophage cell line [20]. In contrast, H-89 suppresses LPS-induced COX-2 expression in RAW264.7 cells [21], These results are consistent to our results with HGFs.

Next, we discuss whether aminophylline at a concentration used in clinic enhances the inflammatory response in periodontal disease. The serum concentrations of theophylline in the therapeutic range are 8 to 20 μg/ml [6,22,23]. Concentrations of theophylline above 20 μg/ml lead to adverse effects such as arrhythmia, convulsion, and central nervous symptoms, and concentrations above 60 μg/ml lead to death [6]. The therapeutic range of aminophylline is considered to be similar to that of theophylline, because aminophylline is a dimer of theophylline. In the present study, up to 10 μg/ml of aminophylline did not affect LPS-induced IL-6, IL-8, or PGE_2 _production. Moreover, although 100 μg/ml of aminophylline enhanced LPS-induced production of these molecules, this concentration of aminophylline is fatal. Therefore, it is unlikely that aminophylline enhances the inflammatory response and exacerbates periodontal disease. Moreover, we conclude that only aminophylline among the drugs we tested does not induce inflammatory responses.

## Conclusion

We demonstrated that the PKA pathway, and also the PKA-activating drugs aminophylline and adrenaline, enhance LPS-induced IL-6, IL-8, and PGE_2 _production by HGFs. However, aminophylline had no relevance to the production of these molecules at a concentration used in clinic (or the therapeutic range). These results suggest that aminophylline does not affect inflammatory responses in periodontal disease.

## Materials and methods

### Reagents

PKA inhibitor H-89 (Sigma, St. Louis, MO) was dissolved in dimethyl sulfoxide (DMSO, Nacalai Tesque, Kyoto, Japan). The cAMP analog dibutyryl cyclic AMP (dbcAMP) and adrenaline [(±)-epinephrine] were purchased from Wako (Osaka, Japan), and aminophylline dihydrate from MP Biomedicals (Illkirch, France). LPS from *Porphyromonas gingivalis *381 was provided by Drs. Tatsuji Nishihara and Nobuhiro Hanada (National Institutes of Public Health, Wako, Japan).

### Cells

HGFs were prepared as described previously [24]. In brief, HGFs were prepared from free gingiva during the extraction of an impacted tooth with the informed consent of the subjects who consulted Matsumoto Dental University Hospital. The free gingival tissues were cut into pieces and seeded onto 24-well plates (AGC Techno Glass Co., Chiba, Japan). HGFs were maintained in Dulbecco's modified Eagle's medium (D-MEM; Sigma) containing 10% heat-inactivated fetal calf serum (FCS), 100 units/ml penicillin, and 100 μg/ml streptomycin, at 37°C in a humidified atmosphere of 5% CO_2_. For passage, HGFs were trypsinazed, suspended, and plated onto new cultures in a 1:3 dilution ratio. HGFs were used between 15th to 20th passages in the assays. Although the proliferation rate was slightly slowed at later passages, the apparent morphological changes were not observed. This study was approved by the Ethical Committee of Matsumoto Dental University (No. 0063).

### Cytokine measurement by enzyme-linked immunosorbent assay (ELISA)

HGFs (10,000 cells/well) were seeded in 96-well plates (AGC Techno Glass Co., Chiba, Japan) and incubated in serum-containing medium at 37°C overnight. Then, the cells were treated with various concentrations of drugs in the absence or presence of LPS (10 ng/ml) for 24 h (200 μl each well). In the experiments using H-89, the cells were pretreated with H-89 (0.1, 1, and 10 μM) or an equal volume of DMSO for 60 min, followed by treatment with a combination of LPS (0 or 10 ng/ml) and H-89 (the same concentration as pretreatment) for 24 h. The concentration of each reagent was determined as the high-frequency concentration according to several previous reports. After the culture supernatants were collected, viable cell numbers were measured using WST-8 (Cell Counting Kit-8; Dojindo, Kumamoto, Japan) according to the manufacturer's instructions. The concentrations of IL-6, IL-8, and PGE_2 _in the culture supernatants were measured by ELISA according to the manufacturers' instructions (IL-6 and IL-8, Biosource International Inc., Camarillo, CA; PGE_2_, Cayman Chemical, Ann Arbor, MI), and were adjusted by the number of remaining cells. Data are represented as ng or pg per 10,000 cells (mean ± S.D., n = 3).

### Statistical analysis

Differences between groups were evaluated by the pairwise comparison test corrected with the Holm method (3 + 3 null hypotheses without drug vs. with drugs in the absence or presence of LPS; total 6 null hypotheses). All computations were performed with the statistical program R [25]. Values with *P *< 0.05 were considered as significantly different.

## Competing interests

The authors declare that they have no competing interests.

## Authors' contributions

TA, HU and HM conceived and designed the experiment. TA and YF performed the experiments. HU performed statistical analyses. KH and TH contributed reagents, materials and analysis tools. TA and HM wrote the paper. All authors read and approved the final manuscript.

## References

[B1] Bartold PM Haynes DR Interleukin-6 production by human gingival fibroblasts J Periodontal Res 1991 26 339 345 10.1111/j.1600-0765.1991.tb02072.x 1831501

[B2] Takada H Mihara J Morisaki I Hamada S Induction of interleukin-1 and -6 in human gingival fibroblast cultures stimulated with *Bacteroides *lipopolysaccharides Infect Immun 1991 59 295 301 1702762 10.1128/iai.59.1.295-301.1991 PMC257740

[B3] Okada H Murakami S Cytokine expression in periodontal health and disease Crit Rev Oral Biol Med 1998 9 248 266 10.1177/10454411980090030101 9715365

[B4] Noguchi K Ishikawa I The roles of cyclooxygenase-2 and prostaglandin E_2 _in periodontal disease Periodontol 2007 43 85 101 10.1111/j.1600-0757.2006.00170.x 17214837

[B5] Kamemoto A Ara T Hattori T Fujinami Y Imamura Y Wang PL Macrolide antibiotics like azithromycin increase lipopolysaccharide-induced IL-8 production by human gingival fibroblasts Eur J Med Res 2009 14 309 314 10.1186/2047-783X-14-7-309 19661014 PMC3458641

[B6] Hendeles L Weinberger M Theophylline. A "state of the art" review Pharmacotherapy 1983 3 2 44 6344032 10.1002/j.1875-9114.1983.tb04531.x

[B7] Tamura M Tokuda M Nagaoka S Takada H Lipopolysaccharides of *Bacteroides intermedius *(*Prevotella intermedia*) and *Bacteroides *(*Porphyromonas*) *gingivalis *induce interleukin-8 gene expression in human gingival fibroblast cultures Infect Immun 1992 60 4932 4937 1328062 10.1128/iai.60.11.4932-4937.1992 PMC258250

[B8] Sismey-Durrant HJ Hopps RM Effect of lipopolysaccharide from *Porphyromonas gingivalis *on prostaglandin E_2 _and interleukin-1β release from rat periosteal and human gingival fibroblasts *in vitro* Oral Microbiol Immunol 1991 6 378 380 10.1111/j.1399-302X.1991.tb00510.x 1668251

[B9] Ara T Kurata K Hirai K Uchihashi T Uematsu T Imamura Y Furusawa K Kurihara S Wang PL Human gingival fibroblasts are critical in sustaining inflammation in periodontal disease J Periodontal Res 2009 44 21 27 10.1111/j.1600-0765.2007.01041.x 19515019

[B10] Ara T Fujinami Y Imamura Y Wang PL Lipopolysaccharide-treated human gingival fibroblasts continuously produce PGE-2 J Hard Tissue Biol 2008 17 121 124 10.2485/jhtb.17.121

[B11] Lohrer P Gloddek J Nagashima AC Korali Z Hopfner U Pereda MP Arzt E Stalla GK Renner U Lipopolysaccharide directly stimulates the intrapituitary interleukin-6 production by folliculostellate cells via specific receptors and the p38α mitogen-activated protein kinase/nuclear factor-κB pathway Endocrinology 2000 141 4457 4465 10.1210/en.141.12.4457 11108255

[B12] Song J Duncan MJ Li G Chan C Grady R Stapleton A Abraham SN A novel TLR4-mediated signaling pathway leading to IL-6 responses in human bladder epithelial cells PLoS Pathog 2007 3 541 552 10.1371/journal.ppat.0030060 PMC1857715 17465679

[B13] Cho IJ Woo NR Shin IC Kim SG H89, an inhibitor of PKA and MSK, inhibits cyclic-AMP response element binding protein-mediated MAPK phosphatase-1 induction by lipopolysaccharide Inflamm Res 2009 58 863 872 10.1007/s00011-009-0057-z 19547917

[B14] Tang Y Feng Y Wang X Calcitonin gene-related peptide potentiates LPS-induced IL-6 release from mouse peritoneal macrophages J Neuroimmunol 1998 84 207 212 10.1016/S0165-5728(97)00257-9 9628464

[B15] Geng Y Zhang B Lotz M Protein tyrosine kinase activation is required for lipopolysaccharide induction of cytokines in human blood monocytes J Immunol 1993 151 6692 6700 8258685

[B16] Farmer P Pugin J β-adrenergic agonists exert their "anti-inflammatory" effects in monocytic cells through the IκB/NF-κB pathway Am J Physiol Lung Cell Mol Physiol 2000 279 L675 L682 11000127 10.1152/ajplung.2000.279.4.L675

[B17] Zhong WW Burke PA Drotar ME Chavali SR Forse RA Effects of prostaglandin E-2, cholera toxin and 8-bromo-cyclic AMP on lipopolysaccharide-induced gene expression of cytokines in human macrophages Immunology 1995 84 446 452 7751029 PMC1415127

[B18] Yoshimura T Kurita C Nagao T Usami E Nakao T Watanabe S Kobayashi J Yamazaki F Tanaka H Inagaki N Nagai H Inhibition of tumor necrosis factor-α and interleukin-1β production by β-adrenoceptor agonists from lipopolysaccharide-stimulated human peripheral blood mononuclear cells Pharmacology 1997 54 144 152 10.1159/000139481 9127437

[B19] Hinz B Brune K Pahl A Cyclooxygenase-2 expression in lipopolysaccharide-stimulated human monocytes is modulated by cyclic AMP, prostaglandin E-2, and nonsteroidal anti-inflammatory drugs Biochem Biophys Res Commun 2000 278 790 796 10.1006/bbrc.2000.3885 11095985

[B20] Lo CJ Fu M Lo FR Cryer HG Cyclooxygenase 2 (COX-2) gene activation is regulated by cyclic adenosine monophosphate Shock 2000 13 41 45 10.1097/00024382-200013010-00008 10638668

[B21] Caivano M Cohen P Role of mitogen-activated protein kinase cascades in mediating lipopolysaccharide-stimulated induction of cyclooxygenase-2 and IL-1β in RAW264 macrophages J Immunol 2000 164 3018 3025 10706690 10.4049/jimmunol.164.6.3018

[B22] Koup JR Schentag JJ Vance JW Kuritzky PM Pyszczynski DR Jusko WJ System for clinical pharmacokinetic monitoring of theophylline therapy Am J Hosp Pharm 1976 33 949 956 790952

[B23] Mitenko PA Ogilvie RI Rational intravenous doses of theophylline N Engl J Med 1973 289 600 603 10.1056/NEJM197309202891202 4723589

[B24] Nakazono Y Ara T Fujinami Y Hattori T Wang PL Preventive effects of a kampo medicine, hangeshashinto on inflammatory responses in lipopolysaccharide-treated human gingival fibroblasts J Hard Tissue Biol 2010 19 43 50 10.2485/jhtb.19.43 20410594

[B25] R Development Core Team R: A Language and Environment for Statistical Computing 2011 http://www.R-project.org/[ISBN 3-900051-07-0]

